# Medical Evacuations out of U.S. Central and Africa Commands Among the Active and Reserve Components of the U.S. Armed Forces, 2023

**Published:** 2024-07-20

**Authors:** Armed Forces Health Surveillance Division Defense Health Agency, US

## Abstract

**What are the new findings?:**

Mental health disorders and injuries were the most common diagnostic categories in 2023 among service members medically evacuated from U.S. Central Command (CENTCOM) and U.S. Africa Command (AFRICOM). In 2023, 724 service members were medically evacuated from CENTCOM and 225 were evacuated from AFRICOM, with hospitalization required for 197 (27.2%) and 50 (22.2%), respectively. Most service members who were medically evacuated from CENTCOM or AFRICOM were returned to full duty status following their post-evacuation hospitalizations or outpatient evaluations.

**What is the impact on readiness and force health protection?:**

In 2023, evacuations for both battle and non-battle injuries from U.S. CENTCOM increased, following a period of decline. The number of service members medically evacuated in 2023 from AFRICOM remained unchanged from the previous year.

## 1 BACKGROUND

This report summarizes the nature, numbers, and trends of conditions for which military members were medically evacuated from the U.S. Central Command (CENTCOM) or Africa Central Command (AFRICOM) operations during 2023, with historical comparisons to the previous 4 years. During deployed military operations, initial medical care is provided by military medical personnel stationed within the operational theater, but some injuries and illnesses require medical care outside the theater of operation. In such cases, affected individuals are usually transported to a permanent military medical facility, usually in Europe or the U.S., for definitive diagnosis or care. Because medical evacuations are resource-intensive, they are employed for serious medical conditions, some of which are directly related to participation in, or support of, military operations. Other medical conditions that are unrelated to operational activities but necessitate medical evacuation may be preventable.

With completion of the withdrawal of all U.S. military forces from Afghanistan on August 31, 2021, followed by the conclusion of the U.S. combat mission in Iraq on December 9, 2021,^1,2^ U.S. military operations were substantially reduced in the CENTCOM area of responsibility (AOR). To sustain counterterrorism operation successes, force deployment continues in all AORs, in addition to assistance, advice, and accompaniment of selected partners’ security forces.^3^

This report only includes medical evacuations from CENTCOM and AFRICOM, without describing any medical evacuations from recent troop deployment to the U.S. European Command (EUCOM), U.S. Indo-Pacific Command (INDOPACOM), or U.S. Southern Command (SOUTHCOM). *MSMR* has historically reported medical evacuations from CENTCOM due to large numbers of service members deployed for named operations such as Operation Iraqi Freedom, Operation Enduring Freedom, and Operation New Dawn. The AFRICOM AOR was added to this annual report in 2021 due counterterrorism force deployment.^3^ Future reports may review medical evacuations from other AORs, as required by leadership interest or changing operational tempos.

## 2 METHODS

The surveillance population for this analysis included all members of the active and reserve components of the U.S. Army, Navy, Air Force, Marine Corps, and Space Force deployed to the CENTCOM or AFRICOM AORs for any length of time from January 1, 2019 through December 31, 2023. Medical evacuations by the U.S. Transportation Command (TRANSCOM) from the CENTCOM or AFRICOM AORs to a medical treatment facility outside the operational theater were assessed from records maintained in the TRANSCOM Regulating and Command and Control Evacuation System (TRAC2ES). Inclusion criteria for this analysis required that any medical evacuee have at least 1 inpatient or outpatient medical encounter at a permanent military medical facility in the U.S. or Europe within an interval of 5 days preceding to 10 days following a reported evacuation date. CENTCOM and AFRICOM evacuation data are presented separately.

Medical evacuations were classified by the cause and nature of the precipitating medical condition, based on information in relevant evacuation and medical encounter records. All medical evacuations were classified as “battle injuries” or “non-battle injuries and illnesses,” based on entries in the TRAC2ES evacuation record.

Evacuations due to non-battle injuries and illnesses were further classified into 18 illness and injury categories based on International Classification of Diseases, 9th and 10th Revisions (ICD-9 and ICD-10, respectively) diagnostic codes reported in medical encounter records following evacuation. All records of hospitalizations and ambulatory visits from 5 days preceding until 10 days following the reported date of each medical evacuation were identified from Defense Medical Surveillance System (DMSS) data. The primary (first-listed) diagnosis for either hospitalization or earliest ambulatory visit after evacuation was used to classify the condition that necessitated the evacuation. If the first-listed diagnostic code specified an external cause of injury (ICD-9 E-code or ICD-10 V-, W-, X-, or Y-code) or an encounter for a condition other than a current illness or injury, the secondary diagnosis specifying illness or injury (ICD-9: 001-999 or ICD-10: A00-T88, U07.1, U09.9) was used. If no secondary diagnosis was provided, or if the secondary diagnosis also was an external cause code, the first-listed diagnostic code of a subsequent encounter was used.

## 3 RESULTS

In 2023, the CENTCOM AOR required 724 medical evacuations, while 225 medical evacuations originated from the AFRICOM AOR. Evaluation of the subsequent hospitalizations and ambulatory visits outside the operational theater during the requisite period following evacuation indicates that mental health disorders accounted for the most medical evacuations from CENTCOM (n=199; 27.5%), while non-battle injuries accounted for the most medical evacuations from AFRICOM (n=51; 22.7%) (**Table 1**). Most medical evacuations from CENTCOM (88.1%) and AFRICOM (87.6%) were assigned routine precedence.

The annual numbers of CENTCOM medical evacuations attributable to battle injuries remained stable in 2019 (n=59) and 2020 (n=59), and substantially decreased in 2021 (n=7), 2022 (n=3), and 2023 (n=14) (data not shown), following the conclusion of major combat operations.^1,2^ Annual CENTCOM medical evacuations attributable to disease and non-battle injuries also declined during the 5-year surveillance period, from 1,077 disease and non-battle injuries in 2019 to 710 disease and non-battle injuries in 2023 (**Figure**). Annual medical evacuations from AFRICOM that were attributable to battle injuries peaked at 6 in 2019, falling to 4 in 2020 and then 0 in 2021, with a rise to 2 in 2022 before falling to 0 again in 2023 (data not shown). Notably, the annual numbers of AFRICOM medical evacuations attributable to non-battle injuries and diseases in 2022 (n=225) and 2023 (n=225) exceeded the previous 3 years but remained much lower than CENTCOM (**Figure**).

**Demographic and military characteristics**

Non-battle injuries were the leading diagnostic categories of evacuations of male service members from CENTCOM and AFRICOM in 2023. Mental disorders were the leading diagnoses for female evacuees from both AORs, with the addition of non-battle injuries and signs, symptoms and ill-defined conditions in AFRICOM (**Table 1**). Compared to men, female service members in CENTCOM and AFRICOM had a higher proportion of medical evacuations for mental health disorders and genitourinary system disorders (**Table 1**). Male service members from both AORs, in contrast, had higher proportions of evacuation for non-battle injuries, musculoskeletal system disorders, and digestive system conditions.

The largest numbers and proportions of evacuees from CENTCOM and AFRICOM involved non-Hispanic White service members, those aged 20-24 years, members of the Army, and senior and junior enlisted personnel (**Table 2**).

**Most frequent specific diagnoses**

Among men and women in both AORs, the leading 3-digit ICD-10 code for mental health disorders indicated reaction to severe stress and adjustment disorders (F43) (**Table 3**). This ICD-10 code represented over two-thirds of the mental disorder diagnoses among men and women in both AORs (data not shown).

Over 10% of all medical evacuations in both AORs were attributed to signs, symptoms and ill-defined conditions (R00-R99) (**Table 1**). The primary diagnoses for the R00-R99 major diagnostic category were not clustered around 1 diagnosis but were diffused throughout this ICD-10 code chapter (data not shown).

**Disposition**

Hospitalizations were required for 197 (27.2%) of the medical evacuees from CENTCOM (n=724) and 50 (22.2%) from AFRICOM (n=225) in 2023 (data not shown).

## 4 DISCUSSION

In 2023, only 14 (1.9%) medical evacuations from CENTCOM and none (0) from AFRICOM were associated with battle injuries in TRAC2ES records. While CENTCOM medical evacuations attributable to non-battle injuries from 2022 to 2023 remained substantially lower than the first 3 years of the surveillance period, AFRICOM non-battle injury medical evacuations remained at their highest level in 2023. These trends coincide with the reduction in forces from CENTCOM and re-establishment of persistent military forces throughout East Africa.3

The leading diagnoses of AFRICOM non-battle injuries were not clustered around any specific ICD-10 code but distributed among diagnoses such as dislocation and sprain of joints and knee ligaments, intracranial injuries, and wrist or hand fractures. This heterogeneity of injury type may be due to the large proportion due to occupational hazards in the deployed environment. Classification by cause of injury rather than affected body system may be more appropriate for this population; the ICD chapter for external causes of morbidity codes is intended for secondary coding purposes and is not mandatory, however. Consequently, completeness and specificity of these external cause codes for injury-related diagnoses may vary according to coding practices.^4,5^

The proportion of CENTCOM medical evacuations (27.5%; n=199) that were attributed to mental health disorders in 2023 represents a decline after increasing proportional trends reported in 2019 (26.8%; n=305), 2020 (27.1%; n=323), 2021 (33.4%; n=322), and 2022 (38.6%, n=266). The proportions of medical evacuation due to mental health disorders are considerably higher than the proportion (11.6%; n=5,892) described by a *MSMR* report examining evacuations from Iraq during a 9-year period between 2003 and 2011.^6,7,8,9^

Several important limitations should be considered when interpreting these results. Demographic data for the deployed population, i.e., person-time for individuals eligible for medical evacuation, are not readily available. The lack of deployed individual person-time precludes calculation of stratified and overall rates for medical evacuations.

Most causes of medical evacuations were estimated for this report from primary (first-listed) diagnoses in DMSS recorded during hospitalizations or initial outpatient encounters following evacuation. Diagnoses recorded in theater through the Theater Medical Data Store (TMDS) are not reflected in this analysis. In some cases, clinical evaluations at fixed medical treatment facilities following medical evacuation may have ruled out serious conditions clinically suspected while in theater, resulting in possible misclassification errors.

Battle injuries rely on proper classification in the TRAC2ES system. Misclassification errors may occur, and given the small number of battle injuries, any misclassification will have a disproportionate effect.

## Figures and Tables

**Table 1:** Numbers and Percentages of Medical Encounters Following Medical Evacuation for Disease and Non-Battle Injuries^a^ from Theater by Area of Responsibility and Major ICD-10 Diagnostic Category, U.S. Armed Forces, 2023

	CENTCOM						AFRICOM					
	Total		Men		Women		Total		Men		Women	
Major Diagnostic Category (ICD-10 codes)	No.	%	No.	%	No.	%	No.	%	No.	%	No.	%
Mental disorders (ICD-10: F01 - F99)	199	27.5	152	25.4	47	37.6	27	12.0	21	11.2	6	16.2
Non-battle injury and poisoning (ICD-10: S00–T88, DOD0101–DOD0105)	183	25.3	170	28.4	13	10.4	51	22.7	45	23.9	6	16.2
Signs, symptoms and ill-defined conditions (ICD-10: R00–R99)	89	12.3	68	11.4	21	16.8	30	13.3	24	12.8	6	16.2
Musculoskeletal system (ICD-10: M00–M99)	83	11.5	74	12.4	9	7.2	30	13.3	28	14.9	2	5.4
Digestive system (ICD-10: K00–K95)	50	6.9	44	7.3	6	4.8	25	11.1	22	11.7	3	8.1
Nervous system and sense organs (ICD-10: G00–G99, H00–H95)	24	3.3	19	3.2	5	4.0	21	9.3	17	9.0	4	10.8
Circulatory system (ICD-10: I00–I99)	21	2.9	19	3.2	2	1.6	6	2.7	6	3.2	0	0.0
Genitourinary system (ICD-10: N00–N99)	17	2.3	9	1.5	8	6.4	11	4.9	6	3.2	5	13.5
Battle injury (TRAC2ES records)	14	1.9	11	1.8	3	2.4	0	0.0	0	0.0	0	0.0
Neoplasms (ICD-10: C00–D49)	9	1.2	7	1.2	2	1.6	3	1.3	2	1.1	1	2.7
Other (ICD-10: Z00–Z99, except pregnancy-related)	9	1.2	6	1.0	3	2.4	10	4.4	8	4.3	2	5.4
Skin and subcutaneous tissue (ICD-10: L00–L99)	8	1.1	7	1.2	1	0.8	4	1.8	4	2.1	0	0.0
Endocrine, nutrition, immunity (ICD-10: E00–E89)	5	0.7	5	0.8	0	0.0	0	0.0	0	0.0	0	0.0
Pregnancy and childbirth (ICD-10: O00–O9A, relevant Z codes)	5	0.7	0	0.0	5	4.0	0	0.0	0	0.0	0	0.0
Respiratory system (ICD-10: J00–J99, U07.0)	4	0.6	4	0.7	0	0.0	5	2.2	4	2.1	1	2.7
Infectious and parasitic diseases (ICD-10: A00–B99)	3	0.4	3	0.5	0	0.0	2	0.9	1	0.5	1	2.7
Hematologic disorders (ICD-10: D50–D89)	1	0.1	1	0.2	0	0.0	0	0.0	0	0.0	0	0.0
Congenital anomalies (ICD-10: Q00–Q99)	0	0.0	0	0.0	0	0.0	0	0.0	0	0.0	0	0.0
COVID-19 (U07.1, U09.9)	0	0.0	0	0.0	0	0.0	0	0.0	0	0.0	0	0.0
Total	724	100.0	599	100.0	125	100.0	225	100.0	188	100.0	37	100.0

Abbreviations: ICD, International Classification of Diseases, 10th Revision; No., number; TRAC2ES, U.S. Transportation Command (TRANSCOM) Regulating and Command and Control Evacuation System; COVID-19, coronavirus disease 2019.

^a^ Classified as Disease and Non-Battle Injuries from injury_type field in TRAC2ES.

**Figure:**
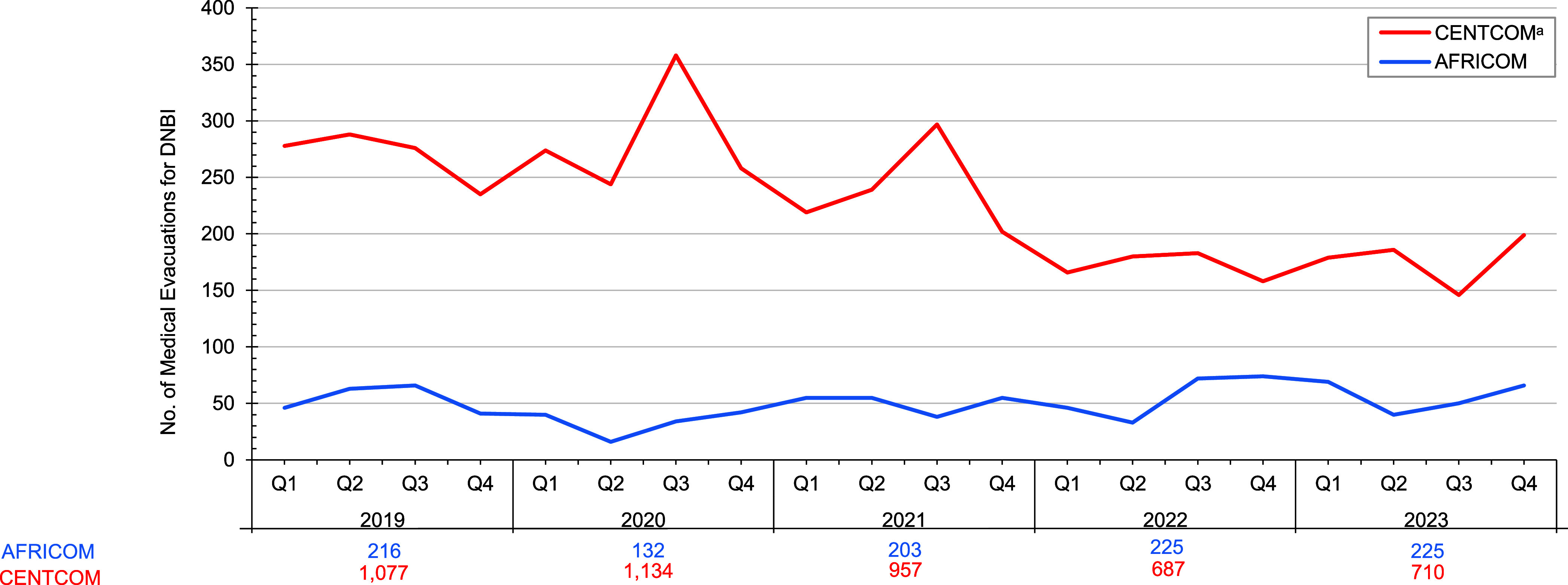
Numbers of Medical Evacuations of U.S. Service Members for Disease and Non-Battle Injuries by Area of Responsibility and Yearly Quarter, 2019–2023^a^

**Table 2:** Demographic and Military Characteristics of Active Component Service Members Medically Evacuated from U.S. Central and Africa Commands, U.S. Armed Forces, 2023

	CENTCOM		AFRICOM	
	No.	% Total	No.	% Total
Total	724	100.0	225	100.0
**Sex**				
Male	599	82.7	188	83.6
Female	125	17.3	37	16.4
**Age group, y**				
<20	14	1.9	2	0.9
20–24	214	29.6	46	20.4
25–29	161	22.2	45	20.0
30–34	132	18.2	49	21.8
35–39	95	13.1	37	16.4
40–44	56	7.7	27	12.0
45+	52	7.2	19	8.4
**Race and ethnicity**				
White, non-Hispanic	400	55.2	129	57.3
Black, non-Hispanic	131	18.1	39	17.3
Hispanic	124	17.1	32	14.2
Other/unknown	69	9.5	25	11.1
**Service branch**				
Army	511	70.6	128	56.9
Navy	69	9.5	34	15.1
Air Force	131	18.1	54	24.0
Marine Corps	13	1.8	9	4.0
**Component**				
Active	335	46.3	70	31.1
Reserve/Guard	389	53.7	155	68.9
**Rank, grade**				
Junior enlisted (E1–E4)	300	41.4	66	29.3
Senior enlisted (E5–E9)	318	43.9	115	51.1
Junior officer (O1–O3; W1–W3)	67	9.3	27	12.0
Senior officer (O4–O10; W4–W5)	39	5.4	17	7.6
**Military occupation**				
Combat-specific^a^	156	21.5	53	23.6
Motor transport	25	3.5	2	0.9
Pilot/aircrew	14	1.9	7	3.1
Repair/engineering	195	26.9	56	24.9
Communications/intelligence	152	21.0	49	21.8
Health care	61	8.4	14	6.2
Other/unknown	121	16.7	44	19.6
**Marital status**				
Married	342	47.2	92	40.9
Single, never married	326	45.0	113	50.2
Other/unknown	56	7.7	20	8.9
**Education level**				
High school or less	425	58.7	107	47.6
Some college	99	13.7	39	17.3
College	163	22.5	62	27.6
Other/unknown	37	5.1	17	7.6
**Precedence^b^**				
Routine	638	88.1	197	87.6
Priority	68	9.4	14	6.2
Urgent	18	2.5	14	6.2
**Transport mode**				
Military	389	53.7	42	18.7
Commercial	31	4.3	7	3.1
Other/unknown	304	42.0	176	78.2

Abbreviations: CENTCOM, Central Command; AFRICOM, Africa Command; No., number; y, years.

^a^ Infantry/artillery/combat engineering/armor.

^b^ Data field within U.S. Transportation Command (TRANSCOM) Regulating and Command and Control Evacuation System (TRAC2ES).

**Table 3:** Most Frequent 3-Digit ICD-10 Diagnoses Associated with Medical Evacuations by Area of Responsibility and Sex, U.S. Armed Forces, 2023

**CENTCOM**					
**Men**			**Women**		
3-Digit ICD-10 Code	Description	No.	3-Digit ICD-10 Code	Description	No.
F43	Reaction to severe stress, and adjustment disorders	120	F43	Reaction to severe stress, and adjustment disorders	37
M54	Dorsalgia	25	M54	Dorsalgia	5
S06	Intracranial injury	20	M25	Other joint disorder, not elsewhere classified	4
M25	Other joint disorder, not elsewhere classified	19	F10	Alcohol-related disorders	3
S46	Injury of muscle, fascia and tendon at shoulder and upper arm level	17	F32	Depressive episode	3
**AFRICOM**					
**Men**			**Women**		
3-Digit ICD-10 Code	Description	No.	3-Digit ICD-10 Code	Description	No.
F43	Reaction to severe stress, and adjustment disorders	16	F43	Reaction to severe stress, and adjustment disorders	4
M54	Dorsalgia	9	R68	Other general symptoms and signs	2
M25	Other joint disorder, not elsewhere classified	7	S82	Fracture of lower leg, including ankle	2
S46	Injury of muscle, fascia and tendon at shoulder and upper arm level	6	S83	Dislocation and sprain of joints and ligaments of knee	2
S83	Dislocation and sprain of joints and ligaments of knee	6	Z02	Encounter for administrative examination	2

Abbreviations: ICD, International Classification of Diseases, 10th Revision; CENTCOM, Central Command; AFRICOM, Africa Command; No., number.
